# Optogenetic Tuning of Ligand Binding to The Human T cell Receptor Using The opto-ligand-TCR System

**DOI:** 10.21769/BioProtoc.3540

**Published:** 2020-03-05

**Authors:** O. Sascha Yousefi, Maximilian Hörner, Maximilian Wess, Vincent Idstein, Wilfried Weber, Wolfgang W. A. Schamel

**Affiliations:** 1 Signalling Research Centres BIOSS and CIBSS, University of Freiburg, University of Freiburg, 79104 Freiburg, Germany; 2 Faculty of Biology, University of Freiburg, 79104 Freiburg, Germany; 3 Center for Chronic Immunodeficiency (CCI), Medical Center Freiburg and Faculty of Medicine, University of Freiburg, 79104 Freiburg, Germany

**Keywords:** Optogenetic, T cell receptor, Reversible ligand binding, Opto-ligand-TCR, Spatiotemporal control

## Abstract

T cells are one major cell type of the immune system that use their T cell antigen receptor (TCR) to bind and respond to foreign molecules derived from pathogens. The ligand-TCR interaction half-lives determine stimulation outcome. Until recently, scientists relied on mutating either the TCR or its ligands to investigate how varying TCR-ligand interaction durations impacted on T cell activation. Our newly created opto-ligand-TCR system allowed us to precisely and reversibly control ligand binding to the TCR by light illumination. This system uses phytochrome B (PhyB) tetramers as a light-regulated TCR ligand. PhyB can be photoconverted between a binding (ON) and non-binding (OFF) conformation by 660 nm and 740 nm light illumination, respectively. PhyB ON is able to bind to a synthetic TCR, generated by fusing the PhyB interacting factor (PIF) to the TCRβ chain. Switching PhyB to the OFF conformation disrupts this interaction. Sufficiently long binding of PhyB tetramers to the PIF-TCR led to T cell activation as measured by calcium influx. Here, we describe protocols for how to generate the tetrameric ligand for our opto-ligand-TCR system, how to measure ligand-TCR binding by flow cytometry and how to quantify T cell activation via calcium influx.

## Background

Life depends to a large extent on the precise spatial and temporal coordination of molecular events. This is particularly important in cellular decision processes, for which cells constantly interpret signals from their environment in order to decide how to respond. Due to the lack of appropriate approaches, the impact of kinetics and localization of signaling processes on cellular decisions is still not well understood. Now the emerging field of optogenetics enables to perform the experiments required to fill this knowledge gap (Kolar and Weber, 2017; Goglia and Toettcher, 2019). As an example, we use T cells stimulated via their T cell antigen receptor (TCR) in this protocol.

T cells are a crucial part of the adaptive immune system. Beyond their role in protecting the body from infections, T cells have recently gotten attention for their potential in cancer immunotherapy. Hence, a better understanding of the mechanisms behind T cell activation is highly wanted (Spear *et al.*, 2019). Using their TCR, T cells can sense foreign particles, such as viruses and bacteria. Those particles are recognized by the TCR in the form of pathogen-derived peptides presented on major histocompatibility complex (MHC) proteins. These peptide-MHC conjugates serve as high affinity ligands for the TCR (Davis *et al.*, 1998). Importantly, self peptides derived from endogenous proteins are presented on MHC as well. Self peptide-MHCs also bind to the TCR, but with low affinity and thus do not result in activatory signaling. It is therefore clear that TCRs are able to distinguish between ligands of different affinity (Holler and Kranz, 2003) and it has been proposed that T cells are able to make this differentiation based on the ligand binding time to the TCR (McKeithan, 1995).

So far, the majority of T cell researchers used peptides with point mutations, presented on MHC to investigate the effect of varying ligand affinity (and indirectly binding time) on T cell activation (Matsui *et al.*, 1991 and 1994; Weber *et al.*, 1992; Sykulev *et al.*, 1994; Corr *et al.*, 1994; Lyons *et al.*, 1996; Daniels *et al.*, 2006). Alternative methods for changing ligand-TCR interaction time are the use of mutated superantigens (Andersen *et al.*, 2001) or the mutation of the TCR itself (Tan *et al.*, 2017) All those approaches have in common that they fail to exclusively manipulate the TCR-ligand binding time without affecting other properties of the binding event, such as on-rate, enthalpy, entropy, geometry of binding, Gibbs energy or ability to withstand forces.

**Figure 1.**
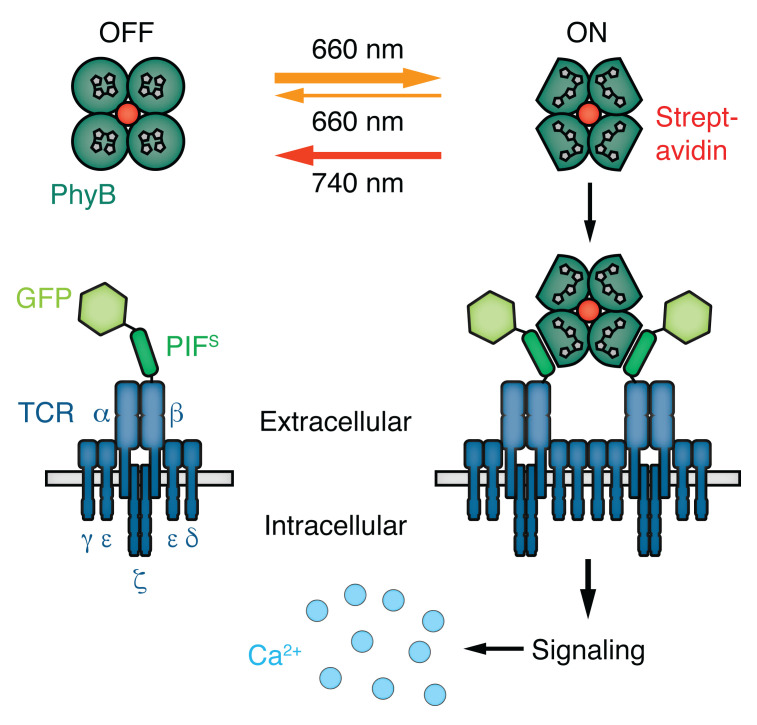
Scheme of the opto-ligand-TCR system. Phytochrome B (PhyB) tetramers can be switched between a binding ON and a non-binding OFF state via 660 nm or 740 nm light illumination, respectively. PhyBt ON are able to bind to Jurkat T cells expressing GFP-PIF^S^-TCR on the surface, thereby activating downstream signaling, part of which is the influx of calcium ions.

To overcome these experimental restrictions we have developed the opto-ligand-TCR system (Yousefi *et al.*, 2019), by making use of the light-dependent interaction between phytochrome B (PhyB) and PhyB interacting factor (PIF) (Levskaya *et al.*, 2009; Toettcher *et al.*, 2013; Kolar and Weber, 2017). We chose the PhyB-PIF system as the optogenetic switch for our system, since it allows for active, light-dependent conformational changes in both directions on a timescale of milliseconds to seconds. On the basis of our expertise to engineer and work with TCRs (Minguet *et al.*, 2007; Swamy *et al.*, 2016; Baeuerle *et al.*, 2019; Schamel *et al.*, 2019), we fused a PIF optimized for the secretory pathway (PIF^S^) together with a monomeric green fluorescent protein (GFP) to the TCRβ chain. This synthetic GFP-PIF^S^-TCRβ was expressed as part of the complete TCR complex on the surface of Jurkat T cells (Figure 1). Biotinylated PhyB molecules were tetramerized via streptavidin and these PhyB tetramers (PhyBt) were used as multimeric TCR ligands. 660 nm light illumination of PhyB led to a switch to the PIF-binding ON state (usually referred to as Pfr state) and 740 nm illumination reverses PhyB to the non-binding OFF state (usually referred to as Pr state) (Mancinelli, 1994). Hence, our opto-ligand-TCR system enabled us to specifically control ligand binding times via light illumination using the same ligand-receptor pair and without introducing mutations to the TCR or its ligands.

Our novel system allows high spatiotemporal control over reversible ligand binding to the TCR. This unique feature of the opto-ligand-TCR system could enable researchers to locally or timely restrict ligand-receptor interaction. Fusing PIF to other receptors would allow to control ligand binding to those receptors as well, as we previously demonstrated for integrins (Baaske *et al.*, 2019). Further, our system could be used to investigate the signaling events that happen after ligand dissociation, which have been mostly neglected due to the lack of appropriate methods.

## Materials and Reagents

1. Pipette tips 0.1-20 µl (VWR, catalog number: 613-1067)
2. Pipette tips 1-200 µl (Carl Roth, catalog number: 7058)
3. Pipette tips 100-1,200 µl (Ratiolab, catalog number: 2400610)
4. 0.22 µm syringe filters (GE Healthcare, Whatman, catalog number: 10462200)
5. 1 ml and 5 ml syringes (Terumo, catalog numbers: SS+01T1 and SS*05LE1)
6. 1.5 ml reaction tubes (Sarstedt, catalog number: 72.690.001)
7. 15 ml and 50 ml conical tubes (Greiner Bio-One, catalog numbers: 188271 and 227261)
8. 3.5 ml FACS tubes (Sarstedt, catalog number: 55.484)
9. HiLoad Superdex 200 pg column (GE Healthcare, catalog number: 28989335), store at 4 °C
10. Jurkat GFP-PIF^S^-TCR cells (Yousefi *et al.*, 2019)
11. Streptavidin, DyLight650-conjugated (Thermo Fisher, Invitrogen, catalog number: 84547), store at 4 °C; molecular weight is approximately 53 kDa
12. Dulbecco’s phosphate-buffered saline (PBS) (Sigma-Aldrich, catalog number D8537), store at 4 °C
13. Tris-(2-carboxyethyl)-phosphine (TCEP) hydrochloride (Carl Roth, catalog number: HN95), store at 4 °C
14. NaN_3_ (Carl Roth, catalog number: K305), store at room temperature
15. Indo-1 (Thermo Fisher, Invitrogen, catalog number: I1223), store at 4 °C
16. Pluronic F-127 (Thermo Fisher, Invitrogen, catalog number: P3000MP), store at room temperature
17. Roswell Park Memorial Institute (RPMI) medium (US Biologicals, catalog number: R9002), store powder at room temperature, store dissolved medium at 4 °C
18. Fetal bovine serum (FBS) (Sigma-Aldrich, catalog number F7524), store at 4 °C
19. 4-(2-hydroxyethyl)-1-piperazineethanesulfonic acid (HEPES) (Thermo Fisher, catalog number: 15630-080), store at 4 °C
20. Purified PhyB-AviTag monomers (Hörner *et al.*, 2020, store at -80 °C; molecular weight is approximately 74 kDa
21. NaOH pellets (Merck, catalog number: 1064981000)
22. 10% NaN_3_ (see Recipes)
23. 5 M NaOH (see Recipes)
24. 0.5 M TCEP (see Recipes)
25. Protein buffer (see Recipes)
26. FACS buffer (see Recipes)
27. Stimulation medium (see Recipes)

## Equipment

1. Pipettes (Eppendorf, catalog number: 3123000900)
2. ÄKTAexplorer 10S (GE Healthcare, ÄKTAexplorer 10S)
3. MACSQuant X Flow Cytometer, customized with 20 mW 355 nm laser and 405/20 nm as well as 530/30 nm emission bandpass filters (Miltenyi Biotec)
4. Centrifuge 5810 R (Eppendorf)
5. Incubator HeraCell 150i (Thermo Fisher)
6. Sterile hood Safe 2020 (Thermo Fisher)
7. pxONE equipped with 660 nm and 740 nm LEDs (Opto Biolabs)
8. Green safe light: Deco flex RGB LED strip, set to green light (Osram, catalog number: 76123)

## Software

1. Unicorn 5.11 (GE Healthcare)
2. FlowJo 9 (Tree Star Inc.)
3. Prism 6 (GraphPad Software Inc.)
4. Illustrator CC (Adobe Inc.)

## Procedure

- Phytochrome B tetramer (PhyBt) production
  1. Purify biotinylated phytochrome B (PhyB-AviTag) monomers as described in Hörner *et al.*, 2020.
  2. Mix a 10-fold molar excess of PhyB-AviTag monomers at a concentration between 1 and 5 mg/ml with DyLight650-conjugated streptavidin in protein buffer for a total volume of 2.5 ml and incubate either for 2 h at room temperature or overnight at 4 °C and in the dark.
  3. Separate PhyB tetramers (PhyBt) from the excess of PhyB-AviTag monomers via size exclusion chromatography using a HiLoad Superdex 200 pg column on an ÄKTAexplorer 10S chromatography system.
    - Perform all steps in a cold room at 4 °C and minimize protein exposure to light. Use a flow rate of 1 ml/min.
    - Equilibrate the column with two column volumes freshly prepared protein buffer.
    - Load the protein mixture on the column using a 2 ml sample loop and run for two column volumes while collecting 1 ml fractions.
    - Protein elution can be monitored by the absorbance at 280 nm (A_280_), PhyB and DyLight650-conjugated streptavidin by the absorbance at 650 nm (A_650_) and PhyB monomers and tetramers by the absorbance at 365 nm (A_365_). See Figure 2 for an example of a purification run.
    - Pool all PhyBt-containing fractions and determine the PhyBt concentration by the ΔΔA method:
      - Perform a spectral analysis of the purified PhyBt as described for PhyB-AviTag monomers in Hörner *et al.*, 2020 under procedure C3.
      - Calculate the ΔΔA value by subtracting the minimum value at 711 nm from the maximum value at 649 nm of the difference spectrum.
      - Multiply the ΔΔA value with 1.1 to get the PhyB concentration in mg/ml. The factor 1.1 was determined from ΔΔA measurements of PhyB-AviTag monomers correlated to protein concentration measurements via Bradford assay.
      - Divide the PhyB concentration in mg/ml by the molecular weight of PhyB-AviTag of 74,000 g/mol to get the PhyB-AviTag concentration in M (mol/L).
      - Divide the the PhyB-AviTag monomer concentration by 4 to get the PhyBt concentration in M.
    - Filter the PhyBt solution using a 0.22 µm syringe filter and aliquot. Sterile aliquots can be stored up to 4 weeks at 4 °C and in the dark.
    - This protocol usually yields 80-90% of the used DyLight650-conjugated streptavidin as PhyBt at a concentration between 0.5 and 3 µM.
      **Figure 2.**
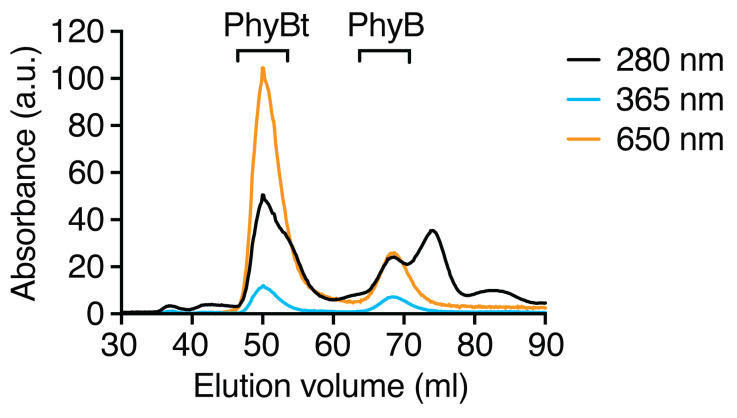
Purification of PhyB tetramers (PhyBt) from PhyB monomers via size exclusion chromatography. PhyB bound to DyLight650-conjugated streptavidin was separated from the excess of PhyB monomers on a HiLoad Superdex 200 pg column. Total protein absorbance was monitored at 280 nm, the absorbance of PhyB was detected at 365 nm and the combined absorbance of PhyB and DyLight650 was followed at 650 nm. Results show one experiment of n > 3.
- Light-dependent PhyBt binding to the GFP-PIF^S^-TCR on the T cell surface
  1. Cultivate Jurkat GFP-PIF^S^-TCR cells according to standard Jurkat cell culture conditions (see *e.g.*, Yousefi *et al.*, 2019). The cells should be kept at a density between 0.5 and 1.0 million cells per ml prior to the experiment.
  2. Transfer 3 x 10^5^ Jurkat GFP-PIF^S^-TCR cells for each sample into FACS tubes.
  3. Centrifuge the samples for 4 min at 300 *× g* at 4 °C.
  4. Perform the following steps on ice (or even better in a 4 °C room) with cold buffers.
  5. Discard the supernatant by aspiration or decanting and resuspend cells in 1 ml FACS buffer.
  6. Repeat the centrifugation and supernatant removal steps as above.
  7. During the centrifugation steps, dilute PhyBt to a final concentration of 100 nM in FACS buffer. The total volume of diluted PhyBt solution depends on the number of samples, with 50 µl being needed per sample.
  8. Divide the PhyBt solution into two and illuminate one half with saturating amounts of 660 nm light, resulting in PhyBt(660), and the other half with saturating amounts of 740 nm light, resulting in PhyBt(740). An illumination for 5 min at 100 µmol/m^2^s is sufficient.
  9. The following handling steps should be performed under green safe light to prevent PhyB photoconversion. It is essential to prevent any white light (sunlight or room light) from hitting the samples.
  10. Resuspend the cell samples in 50 µl of either PhyBt(660) or PhyBt(740) and incubate for 30 min on ice and in the dark.
  11. Wash the samples two times as described under Steps B3-B5.
  12. Resuspend the cells in 200 µl FACS buffer and measure the PhyBt (DyLight650) fluorescence in a flow cytometer. Representative results are depicted in Figure 3.
    **Figure 3.**
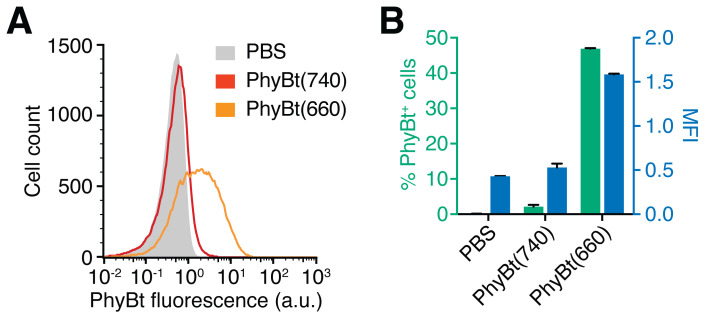
Measurement of PhyBt binding to the GFP-PIF^S^-TCR on the T cell surface. A. Jurkat GFP-PIF^S^-TCR cells were treated with PBS (grey), PhyBt(740) (red) or PhyBt(660) (orange) and surface binding of PhyBt was measured by flow cytometry via DyLight650 fluorescence. Results show one experiment of n > 3 (a.u., arbitrary units). B. Median fluorescence intensity (MFI, blue) and percent of PhyBt-bound cells (green) were quantified as measured in (A). Only PhyBt(660) showed considerable binding to the T cells. Results depict the mean of duplicate measurements ± SD of one experiment out of n > 3.
- Analysis of T cell activation via Ca^2+^ influx upon light-dependent PhyBt binding
  1. Transfer 5 x 10^6^ freshly dividing Jurkat GFP-PIF^S^-TCR cells into a 15 ml conical tube.
  2. Centrifuge the cells for 4 min at 300 *× g* at room temperature.
  3. Discard the supernatant by aspiration and resuspend the cells in 1 ml stimulation medium.
  4. The cells should be resuspended by gentle pipetting and never by vortexing as this can already lead to cell activation and impair the Ca^2+^ influx measurements.
  5. Repeat the centrifugation and supernatant removal as above.
  6. Prepare the Indo-1 staining solution during the centrifugation step by mixing 5 µl Pluronic F-127 with 4 µl Indo-1 and 1 ml stimulation medium.
  7. Resuspend the cells in 1 ml staining solution, transfer the cell suspension into a 1.5 ml reaction tube and incubate for 15 min in a cell culture incubator keeping the lid open.
  8. After 15 min incubation, close the lid of the reaction tube, briefly invert the tube 5-10 times and incubate the cells for another 15 min.
  9. Repeat centrifugation and supernatant removal as in Steps C2 and C3 and resuspend the cells in 500 µl 4 °C cold stimulation medium.
  10. Keep the cells on ice and in the dark for 15 min before starting the measurements.
  11. For each measurement, freshly prepare 1 ml diluted cell suspension by gently mixing 50 µl of the stained cells with 950 µl 37 °C pre-warmed stimulation medium in a FACS tube.
  12. Transfer the FACS tube into the pxONE incubation and illumination device and insert the device into the flow cytometer. Details about the use of the pxONE device can be found on the manufacturer’s website (www.optobiolabs.com) and a video article providing a step-by-step explanation of a prototype is available (Brenker *et al.*, 2016).
  13. Measure 1 min baseline fluorescence, then add between 2 and 200 nM of either PhyBt(660) or PhyBt(740) and measure another 5 min in the dark. Figure 4 depicts an example of such an experiment.
  14. Vary the PhyBt concentration and the illumination conditions to your specific experimental question. Examples of different lighting regimes for various experimental questions can be found in Yousefi *et al.*, 2019.
    **Figure 4.**
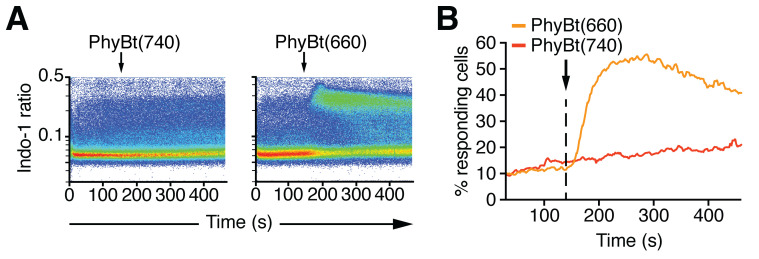
Calcium influx measurement upon light-dependent stimulation with the opto-ligand-TCR system. A. Calcium influx into Jurkat GFP-PIF^S^-TCR cells was measured via Indo-1 fluorescence upon addition of 20 nM PhyBt(740) or PhyBt(660) as indicated. The stimulation was performed in the dark and stimuli addition is indicated by the arrow. Results show one experiment of n > 3. B. The percent of responding cells were quantified from (A) as described in the Data Analysis section. Results show one experiment of n > 3. T cells treated with PhyBt(660) showed calcium influx that peaked at around 250 s as opposed to PhyBt(740)-treated cells.

## Data analysis

The Unicorn software suite was used to control the Äkta chromatography system and analyze the resulting data. FlowJo was used to analyze all flow cytometry data. Only the living cell population was used for the depicted flow cytometry results. Intracellular calcium was quantified by the ratio of Ca^2+^-bound Indo-1 (405/20 nm filter) and Ca^2+^-free Indo-1 (530/30 nm filter). The percentage of responding cells shown in Figure 4A were derived from FlowJo’s kinetics module using the 90^th^ percentile over the baseline measurement between 30 and 60 s. For Figures 2, 3B and 4B, quantified data were exported from Unicorn and FlowJo, respectively, and displayed using Prism. Figures 3A and 4A were directly exported from FlowJo. All figures were compiled using Illustrator.

## Notes

1. Protect PhyB from bright light, like white room light or direct sunlight, by covering it with aluminum foil or dimming the room, if possible. Whenever the conformation of PhyB should not be changed, work under green safe light, which lies at the absorbance minimum of PhyB. White room light or sunlight has a similar effect as 660 nm illumination and converts PhyB to the ON state.
2. PhyB is sensitive to oxidation and therefore should be kept in a reducing buffer by for example degassing solutions and adding 0.5 mM TCEP, β-mercaptoethanol or dithiothreitol. Whenever the experimental conditions prohibit the use of reducing agents, expect and monitor a decline in PhyB to PIF binding activity over the course of several hours.
3. Continuous 660 nm light illumination may prevent PhyBt from binding to the GFP-PIF^S^-TCR as described inYousefi *et al.* (2019). Therefore, use either low 660 nm light intensities or pulsed illumination with dark periods in between. 660 nm light illumination of increasing intensity results in shorter PhyBt binding half-lives to the GFP-PIF^S^-TCR.
4. Due to the photobiology of PhyB, saturating illumination with our 660 nm light source resulted in 80% of the molecules in the PhyB ON state and 20% in the PhyB OFF state. Saturating illumination with our 740 nm light source gave 1% PhyB ON and 99% PhyB OFF. Different light sources with altering emission spectra will result in different PhyB ON:OFF ratios, for details seeSmith *et al.* (2016).

## Recipes

1. 10% NaN_3_
  - Dissolve NaN_3_ in water
  - Store at room temperature
2. 5 M NaOH
  - Dissolve NaOH pellets in water by adding the pellets slowly to the water (exothermic reaction)
  - Store at room temperature
3. 0.5 M TCEP
  - Dissolve TCEP in degassed water
  - Adjust pH to 7.4 with 5 M NaOH
  - Store in 1 ml aliquots at -20 °C
4. Protein buffer
  - PBS
  - Add 0.5 mM TCEP from 0.5 M stock solution
  - Run through 0.22 µm filter and degas
  - Do not store protein buffer, but always prepare freshly
5. FACS buffer
  - PBS
  - 1% FBS
  - 0.02% NaN_3 _(dilute from 10% stock)
  - Store at 4 °C
6. Stimulation medium
  - RPMI
  - 1% FBS
  - 10 mM HEPES
  - Store at 4 °C
